# Novel Coronavirus (2019-nCoV) in Disguise

**DOI:** 10.7759/cureus.7521

**Published:** 2020-04-03

**Authors:** Qian Zhang, Annyella Douglas, Zain U Abideen, Shristi Khanal, Stephanie Tzarnas

**Affiliations:** 1 Internal Medicine, Abington Hospital-Jefferson Health, Abington, USA

**Keywords:** coronavirus disease, covid-19, acute pyelonephritis, novel coronavirus

## Abstract

Novel coronavirus (2019-nCoV) pandemic is currently one of the most influential topics as it not only impacts the field of medicine but most importantly, it affects the lives of many individuals throughout the world. We report an interesting 2019-nCoV case in a tertiary community hospital with the initial concern of acute pyelonephritis without respiratory symptoms that ultimately led to the quarantine of a number of healthcare providers. This case emphasizes the importance of radiological evidence in diagnosing 2019-nCoV in the setting of an initial atypical presentation. It also serves as an example of how healthcare providers may need to increase their suspicion for COVID-19 to ensure self-protection and prompt diagnosis in the era of an ongoing pandemic.

## Introduction

Severe acute respiratory syndrome coronavirus 2 (SARS-CoV-2), previously noted as the 2019 novel coronavirus (2019-nCoV), is a positive-sense single-stranded RNA that was first acknowledged in Wuhan, China that ever since has led to a pandemic spreading over more than 150 different territories across the world [1,2]. Although our understanding of the virus is rapidly evolving, it is currently universally recognized that patients often present with fever and lower respiratory symptoms. Rarely, symptoms may progress to respiratory failure, shock or multiorgan dysfunction. Patients who present to the hospital with fever and respiratory distress along with concurrent risk factors, including advanced age, immunocompromised status, severe comorbidities and recent travel histories, are screened in the emergency department and placed in airborne isolation until the result is negative. However, our case presents a 27-year-old healthy female without past medical history, with a strong suspicion of acute pyelonephritis but ultimately found to have incidental CT findings suspicious for COVID-19. 

## Case presentation

**Figure 1:**
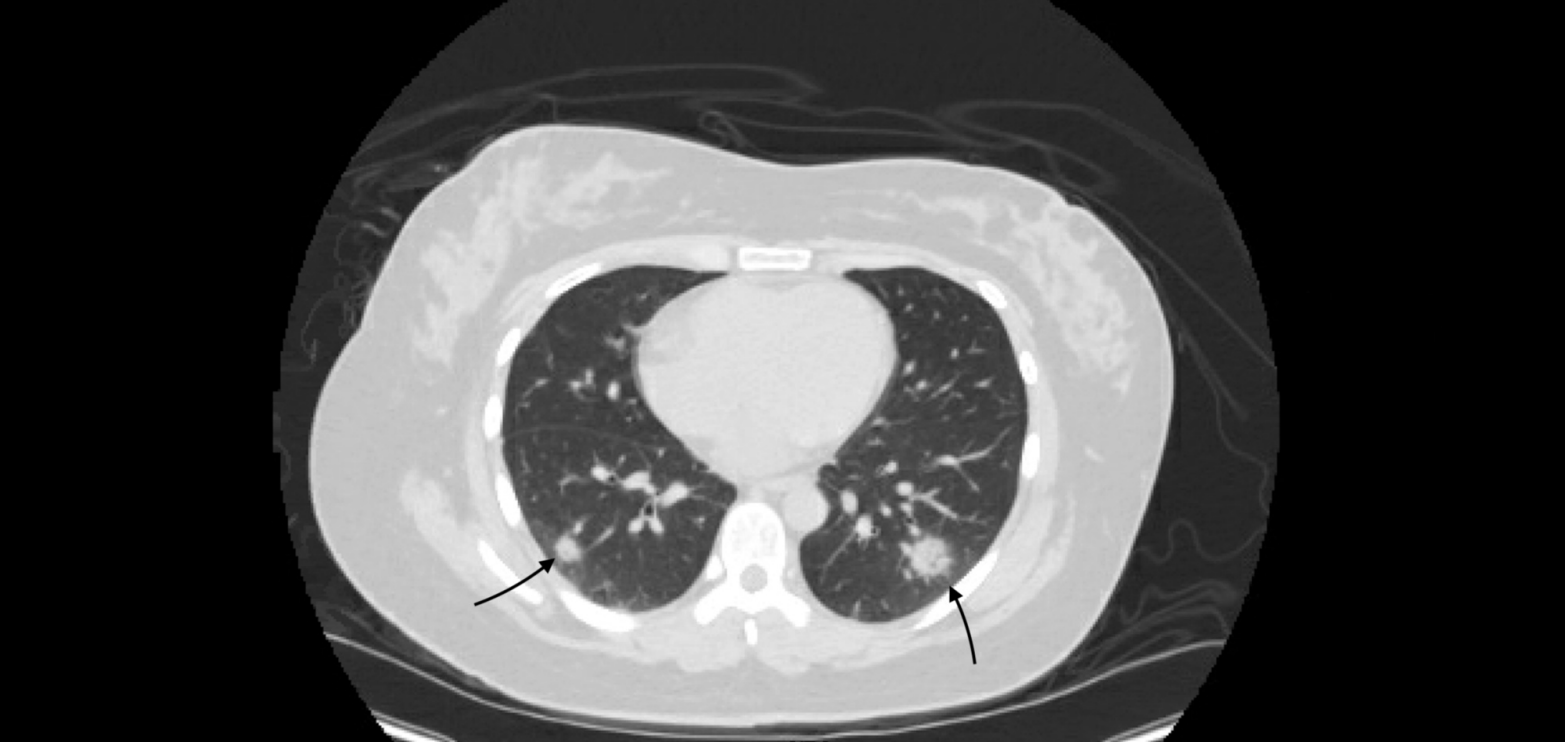
CT of the abdomen and pelvis without contrast revealing multiple cavitating pulmonary nodules

Our patient is a 27-year-old female, who presented with left-sided flank pain. She did not have any past medical history, was a non-smoker and denied recent travel history except for traveling to South Korea in 2018. She worked as a waitress at different restaurants, denied a history of intravenous drug abuse or any recent sick contacts. She had intermittent, non-specific abdominal pain that required two visits to local urgent care centers and was prescribed ibuprofen and tamsulosin. However, her pain worsened the evening prior to admission. The pain was predominantly localized to the left costovertebral angle, associated with subjective fever and dysuria. In the emergency department, her initial vital signs were as follows: temperature 102.5°F, blood pressure 130/80 mmHg, respiratory rate 22 breaths per minute, heart rate 100 beats per minute and oxygen saturation 97% on room air. Physical examination revealed a well-developed, well-nourished woman who was alert and oriented; the heart was regular rate and rhythm without murmurs, rubs or gallops; lungs were clear to auscultation bilaterally without wheeze or rales; non-tender abdomen with normal bowel sounds without masses appreciated; positive left costovertebral angle tenderness; +1 non-pitting edema of the lower extremities. Pertinent laboratory results were as follows: urinalysis positive for large leukocyte esterase with 139 white blood cells, complete blood count showed white blood cells of 4.9 K/UL with absolute lymphocyte of 0.9 K/UL along with platelet counts of 86 K/UL. CT of abdomen and pelvis without contrast was ordered that did not reveal any findings suggestive of acute pyelonephritis, but did incidentally reveal multiple cavitating pulmonary nodules in the visualized lung bases (Figure 1). She was treated with ceftriaxone for urinary tract infection (UTI) with possible underlying left acute pyelonephritis. Moreover, blood cultures were sent for possible sepsis in the setting of the incidental finding of pulmonary nodules found on the CT scan. 

**Figure 2:**
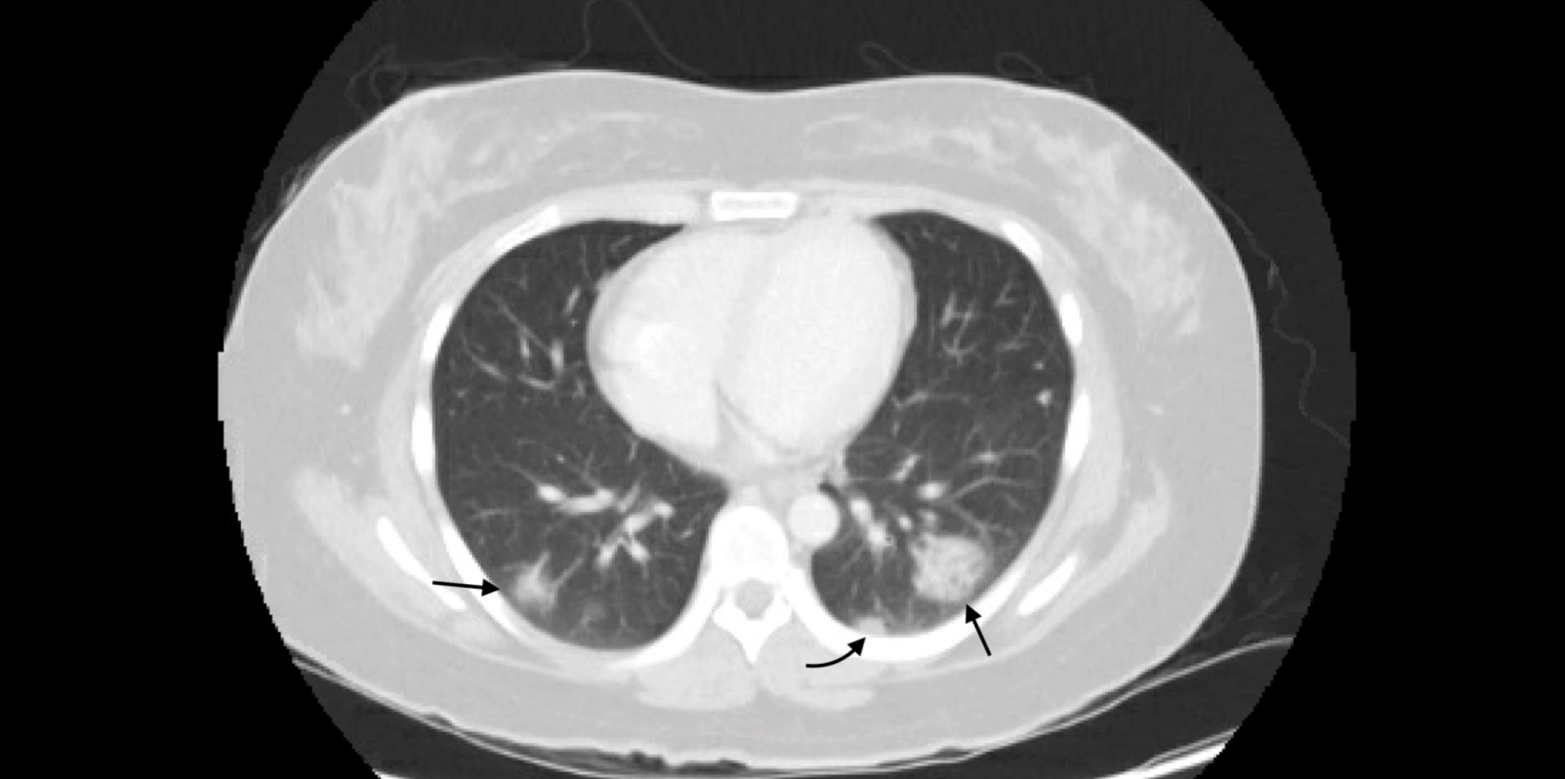
CT of the chest with contrast revealing heterogeneous cavitary opacities at the bilateral lower lobes

On day 2 of admission, the patient reported improvement in flank pain and resolution of her dysuria. She remained febrile with a temperature max (Tmax) of 103.9°F, and blood pressure was noted to decrease. She also became more lethargic and weak. It was determined to broaden antibiotics coverage to vancomycin, cefepime and levofloxacin to empirically treat for possible sepsis due to UTI or acute pyelonephritis. CT of the chest, abdomen and pelvis with contrast was ordered to investigate the incidental findings of bilateral cavitating pulmonary nodules found at the lung bases. The result showed that there was worsening and interval progression of heterogeneous cavitary opacities at the bilateral lower lobes along with more subtle and smaller opacities throughout the remainder of the lungs (Figure 2). There was no evidence and clinical suspicion to believe that these findings correlated with differential diagnosis of an acute inflammatory process such as rheumatoid arthritis or lupus, alveolar hemorrhage syndrome, endovascular infection or atypical pneumonia. She was placed in airborne isolation, and SARS COV-2 was sent to rule out COVID-19 infection due to CT findings. 

On day 4, she remained febrile with Tmax of 102.2°F and temporarily required two liters of nasal cannula due to desaturation to 89%, although she denied feeling short of breath. Her blood pressure remained stable. On day 5, results returned as positive for COVID-19. She developed a significant dry cough. Broad-spectrum antibiotics were discontinued, and she was started on a five-day course of hydroxychloroquine (400 mg BID for one day and then 200 mg BID for four days). Her cough improved over the following days, and two days later, she was subsequently discharged home given her stable clinical status. Tmax upon the day of discharge was 100.8°F. She was instructed to self-quarantine for at least 14 days at the time of symptoms resolution while not on medications. Healthcare providers who had close contact with her without protection were instructed to self-quarantine for 14 days while monitoring for symptoms. A few symptomatic healthcare providers were tested, and results were fortunately negative. 

## Discussion

2019-nCoV is one of the most common differential diagnoses that healthcare providers consider in the current COVID-19 pandemic. The typical symptoms include fever, myalgia, fatigue and lower respiratory symptoms, such as dry cough, dyspnea and respiratory distress. Fever is often one of the most common presenting symptoms [3,4]. Although atypical presentation of COVID-19 is rare, it cannot be excluded. Based on the literature and research, there has been very few documented reports of an atypical presentation of the novel coronavirus. In a case report conducted by Hao et al., a 60-year-old gentleman presented with fatigue but was afebrile along with no lower respiratory tract symptoms [5]. Upon workup for fatigue, CT chest findings demonstrated a patchy high-density shadow in bilateral lungs that gradually improved with time. This prompted testing for COVID-19, and he was found to be positive despite an initial negative oropharyngeal swab. It has overwhelmingly been shown in the literature that an abnormal CT of the chest has >90% sensitivity for COVID-19 [3,4,6-8]. The most common CT chest finding is predominant ground-glass opacity. Other common CT chest findings include air bronchograms, consolidations, interlobular septal thickening and thickening of the adjacent pleura [3]. The usual location of lung involvement is often peripheral and lower lobes.

Similarly, our patient had CT chest findings that were very concerning for COVID-19 infection despite no initial symptoms of lower respiratory symptoms. With the lack of significant risk factors such as advanced age, multiple comorbidities, immunocompromised state or sick contacts in an otherwise 27-year-old healthy female, it was her incidental findings of multiple pulmonary patchy consolidations found on the CT that guided us to suspect COVID-19. However, typical signs that she presented with were fever and supporting laboratory findings of lymphocytopenia and thrombocytopenia throughout her hospitalization course [3,4,6,7].

Healthcare workers are on the frontline for taking care of patients who have possible or confirmed COVID-19. There are guidelines recommended by the Centers for Disease Control and Prevention (CDC) regarding precautions for healthcare workers. To prevent or minimize the spread of infection, patients should be triaged, and extra precautions should be taken for the patients presenting to the hospital with respiratory symptoms. These precautions include placing patients in rooms with doors closed, having patients wear facial masks and wearing appropriate precautions before the patient encounters [9]. However, for patients with unusual presentations such as our patient, it is extremely challenging to initiate adequate prevention right away at the first patient encounter. The best suggestion during this pandemic is to have a high suspicion for all patients, especially those with a fever, as this often signifies an infectious process. Unfortunately, a number of healthcare providers had to be self-quarantined due to lack of awareness in the setting of the atypical presentation. Moreover, hospitals across the nation are actively implementing changes to protect their healthcare providers from possible exposures. It is difficult to ensure bulletproof protection in the setting of pandemic spread especially for healthcare providers battling the virus at the frontline. 

## Conclusions

Physicians should have a high suspicion for COVID-19, especially since there are early atypical presentations. CT chest findings are one of the most helpful ways to aid in diagnosing a patient. However, a healthcare provider’s high clinical suspicion for COVID-19 is even more important prior to ordering imaging studies. Providers should be reminded of the importance of COVID-19 as an essential part when formulating differential diagnoses as there is a lack of literature reviews of atypical presentations. During this ongoing pandemic, this will be important to not only adequately treat patients, but also to increase awareness for proper self-protection.
